# Supplementary material for: Development of a computer-aided design software for the quantitative evaluation of aesthetic damage
Source: PLoS One. 2019 Dec 18;14(12):e0226322. doi: 10.1371/journal.pone.0226322 (PMC6919621; doi:10.1371/journal.pone.0226322)
Supplement: S1 Code site — 1. (ZIP) [file pone.0226322.s001.zip › [EESC_jr][Projeto]Relatorio_07_21/reports/templates_zip/gentelella-master/vendors/starrr/index.html]

Starrr, for jQuery


### Starrr

##### Click to rate:

Your rating was .

##### Advanced:

  


##### Why?

There are other libraries out there, but none that approach the level clarity and conciseness that I wanted. Starrr is less than 75 lines of code -- you can understand the entirety of its source code in about a minute.

##### Dependencies

jQuery.

I'm using Bootstrap + Font Awesome to render the stars, but you're more than welcome to use something else.
